# Retinal thickness changes in different subfields reflect the volume change of cerebral white matter hyperintensity

**DOI:** 10.3389/fneur.2022.1014359

**Published:** 2022-10-17

**Authors:** Xiaohan Lv, Zhenjie Teng, Zhiyang Jia, Yanhong Dong, Jing Xu, Peiyuan Lv

**Affiliations:** ^1^Department of Neurology, Hebei Medical University, Shijiazhuang, China; ^2^Department of Neurology, Hebei General Hospital, Shijiazhuang, China; ^3^Department of Neurology, Hebei Provincial Key Laboratory of Cerebral Networks and Cognitive Disorders, Shijiazhuang, China; ^4^Department of Ophthalmology, Hebei General Hospital, Shijiazhuang, China

**Keywords:** retinal thickness, white matter hyperintensity volume, optical coherence tomography, chronic ischemia, inflammatory response

## Abstract

**Purpose:**

To investigate the relationship between the retinal thickness in different subfields and the volume of white matter hyperintensity (WMH), with the hope to provide new evidence for the potential association between the retina and the brain.

**Methods:**

A total of 185 participants aged over 40 years were included in our study. Magnetic resonance imaging (MRI) was used to image the WMH, and WMH volume was quantitatively measured by a specific toolbox. The thickness of the total retina, the retinal nerve fiber layer (RNFL), and the ganglion cell and inner plexiform layer (GCIP) was measured by optical coherence tomography (OCT) in nine subfields. The association between retinal thickness and WMH volume was demonstrated using binary logistic regression and Pearson correlation analysis.

**Results:**

Participants were divided into two groups by the WMH volume (‰, standardized WMH volume) median. In the quartile-stratified binary logistic regression analysis, we found that the risk of higher WMH volume showed a positive linear trend correlation with the thickness of total retina (95% CI: 0.848 to 7.034; *P* for trend = 0.044)/ GCIP (95% CI: 1.263 to 10.549; *P* for trend = 0.038) at the central fovea, and a negative linear trend correlation with the thickness of nasal inner RNFL (95% CI: 0.086 to 0.787; *P* for trend = 0.012), nasal outer RNFL (95% CI: 0.058 to 0.561; *P* for trend = 0.004), and inferior outer RNFL (95% CI: 0.081 to 0.667; *P* for trend = 0.004), after adjusting for possible confounders. Correlation analysis results showed that WMH volume had a significant negative correlation with superior outer RNFL thickness (*r* = −0.171, *P* = 0.02) and nasal outer RNFL thickness (*r* = −0.208, *P* = 0.004).

**Conclusion:**

It is suggested that central fovea and outer retina thickness are respectively associated with WMH volume. OCT may be a biological marker for early detection and longitudinal monitoring of WMH.

## Introduction

White matter hyperintensity (WMH) is a manifestation of cerebral small vessel disease. It is divided into periventricular white matter hyperintensity (PWMH) and deep white matter hyperintensity (DWMH) according to the location. Its pathology remains unknown. Current studies suggest that it is related to white matter (WM) demyelination, axon loss, and gliosis caused by blood–brain barrier leakage, hypoperfusion, vessel endothelial dysfunction, inflammatory response, oxidative stress, and venous drainage disturbance (1–5). DWMH is more related to ischemic factors and showed more axonal loss, infarction, and tissue loss in postmortem studies. PWMH is closely related to inflammatory factors and showed more edema and gliosis compared to DWMH (6, 7). WMH impairs cognitive function, motor function, mood, and urinary function. It may increase the risk of stroke and dementia, burdening family and society (8). Currently, the clinical evaluation of WMH largely depends on magnetic resonance imaging (MRI). However, MRI is expensive, complicated, and difficult to cooperate with patients, so more economical methods are needed for WMH scans.

The retina is composed of nine retinal neuroepithelial layers [inner limiting membrane, retinal nerve fiber layer (RNFL), ganglion cell layer (GCL), inner plexiform layer (IPL), inner nuclear layer, outer plexiform layer, outer nuclear layer, external limiting membrane, and photoreceptor segments] and retinal pigment epithelial layer (9). Optical coherence tomography (OCT) is a non-invasive imaging technique based on low coherence interferometry that makes it possible to view high-resolution images of the retina *in vivo*. OCT system generates cross-sectional images of the retina that show the reflectivity information captured in depth by means of whiter or darker bands (10). RNFL is composed of ganglion cell axons that travel in bundles until the optic nerve. It is responsible for transmitting visual information to the visual cortex. GCL is composed of the nuclei of ganglion, while IPL is formed by the synaptic connections between ganglion cells, bipolar cells, and amacrine cells (9). The macular fovea is an avascular depressed area in the retinal posterior pole. It is the most visually sensitive part of the retina and is responsible for our ability to read, distinguish colors, and for our spatial resolution capacity (9). In the ophthalmology field, OCT is widely used to detect the early stages of diseases and track the progression after the treatment of retinal pathologies (11).

The retina is regarded to be an extension of the brain. They have the same properties in embryology, physiology, and morphology. First, the retina develops from the diencephalon during the embryonic stage (10). Next, given that ocular arteries descend from the internal carotid artery, the vessels of the retina have many anatomical and physiological traits in common with the vessels of the brain. Researchers have confirmed that retinal vasculopathy and WMH had similar vascular risk factors (12). Furthermore, tight junctions between endothelial cells and peripheral astrocytes, Müller cells, and pericytes build the blood–retinal barrier together, which is reminiscent of the blood–brain barrier (13). The aqueous humor contains abundant immunoregulatory and anti-inflammatory mediators, which resemble the cerebrospinal fluid (CSF) (14). In summary, the retina has similar features to the brain and thus provides a window to explore intracranial lesions.

Optical coherence tomography has been used to explore some cerebral diseases, like Alzheimer's disease (AD), multiple sclerosis, Parkinson's disease, and neuromyelitis optical spectrum disorder (15–18). A previous study found that healthy subjects with a high genetic risk for the development of AD showed early changes in the macular area, where RNFL and IPL were significantly thinner than the control group (15). There is a direct correlation between RNFL thickness and medial temporal lobe volume, especially with the hippocampus volume (19). The decrease of RNFL thickness in the macular area can be observed before the damage to the central nervous memory system, indicating that it may be an early biomarker of AD or cognitive impairment (20). As for the thinner IPL found in the high-risk group of AD, the writer attributed it to the decreased cholinergic activity in this layer (15, 21, 22). Besides, in more advanced stages of AD, ganglion cell and inner plexiform layer (GCIP) complex showed a decrease in thickness in all the subfields compared with the control group (23). Similarly, multiple sclerosis and Parkinson's disease also exhibit accelerated RNFL and GCIP thinning throughout the disease course, particularly in the early stage (16, 17). Neuromyelitis optica spectrum disorder is mainly manifested as the GCIP loss independent of optic neuritis attacks (18). OCT and its related technologies may represent a novel tool to diagnose and distinguish those cerebral diseases (24). According to the above-mentioned studies, we finally selected the thickness of the total retina, RNFL, and GCIP for analysis.

Recent studies have found that retinal changes might be closely related to WMH (25–35). It is known that only retinal vessels can be observed non-invasively *in vivo*. Recent studies have demonstrated the association between WMH and retinal vessel diameter (25), retinopathy (26, 27), altered retinal vessel bifurcation (28) measured by retinal fundus image, and retinal microvascular density measured by optical coherence tomography angiography (OCTA) (29–31), suggesting that microvascular impairment is a cause of WMH. In retinal sublayers, RNFL contains axons, and might reflect the condition of WM. GCIP contains cell bodies and synaptic structures and might reflect the condition of gray matter (GM) (32). Qu et al. found that retinal degeneration in the RNFL and GCIP was independently associated with WMH, and deteriorated with the severity of the WMH (33). A recent study also showed that abnormalities in retinal microvascular density, morphological parameters, and RNFL thickness were correlated with the incidence of moderate-severe WMH (34). Carolina et al. evaluated the WM integrity tract by diffusion tensor imaging and found that GCL and IPL thickness could reflect the whole-brain WM dysfunction in patients with multiple sclerosis (35).

In this study, WMH volume was quantitatively determined, while the retina was divided into nine subfields by OCT. We aim to quantitatively analyze the association between the retinal thickness in different subfields and the volume of WMH to provide new evidence for the potential retina–brain association.

## Materials and methods

### Participants

In this cross-sectional study, we recruited participants from the inpatients of the department of neurology in Hebei General Hospital from October 2019 to April 2022. In total, 185 participants aged over 40 years without dementia underwent complete 3T MRI sequences necessary for evaluating WMH volume and OCT examination necessary for evaluating retinal thickness. Exclusion criteria were as follows: (1) neurological diseases, such as acute and large-area stroke, tumor, epilepsy, mental disorders, central nervous system demyelinating disease, or trauma; (2) any cognitive deterioration history or ever had a diagnosis of dementia; (3) Barthel Activities of Daily Living Index [scale 0–100, 10 items including feeding, personal toileting, bathing, dressing and undressing, getting on and off a toilet, controlling bladder, controlling bowel, moving from wheelchair to bed and returning, walking on level surface (or propelling a wheelchair if unable to walk), and ascending and descending stairs] < 80 (36); (4) contraindications to MRI; (5) primary eye diseases, such as severe diabetic and hypertensive retinopathy, cataract, glaucoma, macular degeneration, intraocular pressure > 21 mmHg, high myopia, or prior ocular surgery; and (6) other hematological and metabolic diseases.

### Clinical assessment

A number of variables, including age, gender, body mass index (BMI), medical history [hypertension, diabetes, coronary heart disease (CHD), and stroke], current smoking, alcohol use, systolic blood pressure (SBP), and diastolic blood pressure (DBP), were collected. We recorded laboratory indicators, including total cholesterol (TC), triglyceride (TG), low-density lipoprotein cholesterol (LDL-C), high-density lipoprotein cholesterol (HDL-C), fasting plasma glucose (FPG), and uric acid (UA).

### Magnetic resonance imaging data acquisition and standardized white matter hyperintensity volume evaluation

All eligible participants underwent 3.0 tesla MR scanners (Signa, GE Healthcare, USA) [T1-weighted, T2-weighted, and fluid-attenuated inversion recovery (FLAIR)] with a slice thickness of 5 mm. WMH is the high signal area on T2-weighted and FLAIR (37).

The WMH was visually evaluated on FLAIR based on the Fazekas scale by an experienced neuro-radiologist. PWMH is WMH contiguous with the margins of each lateral ventricle, while DWMH is WMH separate from the ventricles (38). PWMH score: 0 = absence, 1 = caps or pencil thin lining, 2 = smooth halo, and 3 = irregular distribution with extension into deep WM. DWMH score: 0 = absence, 1 = punctate foci, 2 = beginning confluence of foci, and 3 = large confluent areas.

Structural MRI data were preprocessed by default parameters as implemented in the CAT12 toolbox (Computation Anatomy Toolbox, version 1184, Structural Brain Mapping Group, Jena University Hospital, Germany; http://dbm.neuro.uni-jena.de/cat/) established on SPM12 (Statistical Parametric Mapping 12, Institute of Neurology, London, UK) in Matlab (Matrix Laboratory, version 2021a, The MathWorks, USA) (39). Images were bias-corrected, segmented, and spatially normalized according to the DARTEL algorithm (40). A Gaussian kernel of 8 mm full width at half-maximum was used for the spatial smoothness of MRI data. The CAT12 “check data quality using covariance” procedure was used for quality assurance. We obtained the adjusted volume of GM/ WM/ CSF, and total intracranial volume was determined as the sum of CSF, WM, and GM.

**Figure 1:**
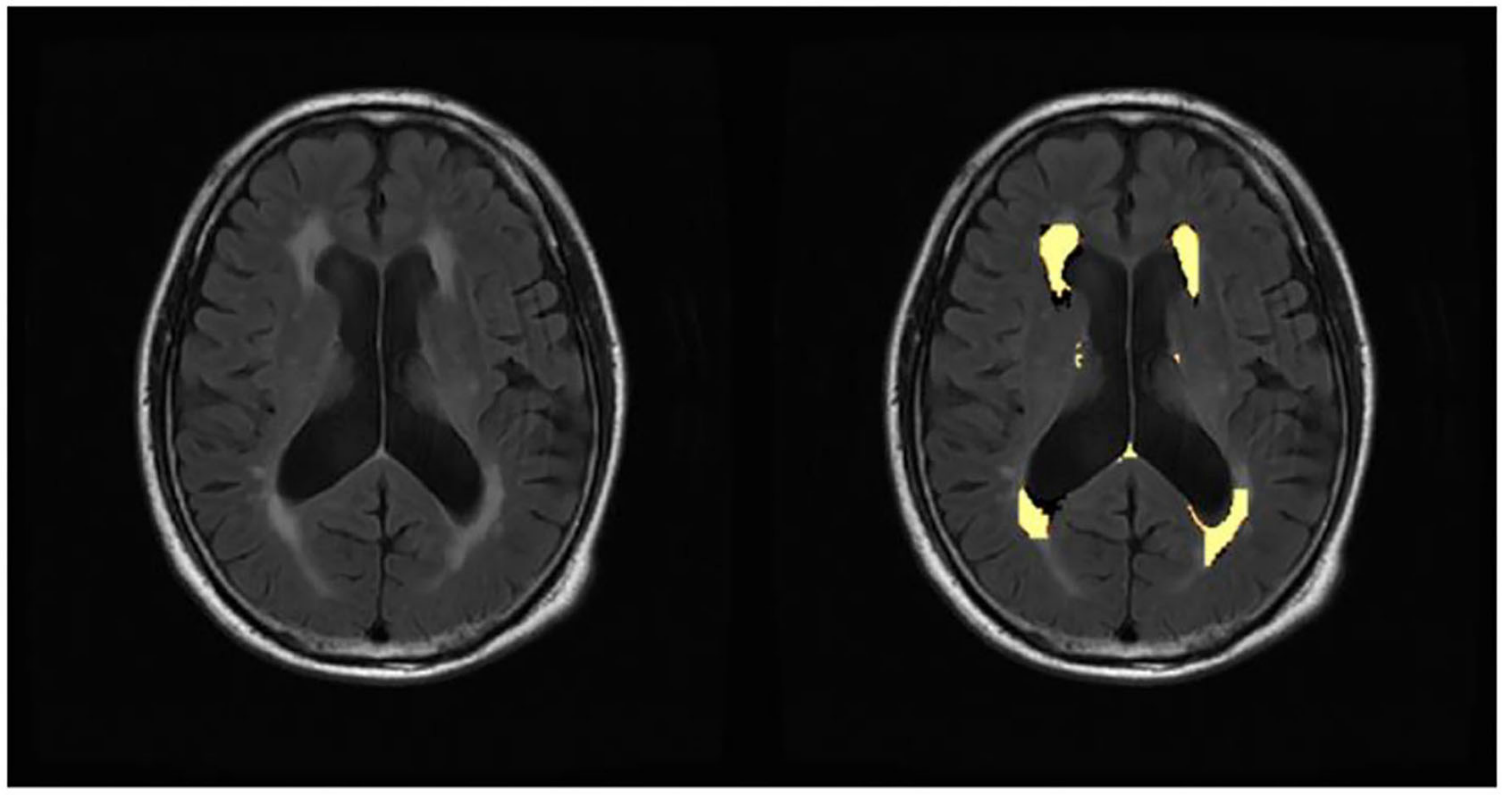
Automated white matter hyperintensity segmentations on sample subject fluid-attenuated inversion recovery scan by lesion growth algorithm.

The WMH volume was segmented by the Lesion Growth Algorithm (LGA) from the LST toolbox (Lesion Segmentation Toolbox, version 3.0.0, Morphometry Group, Department of Neurology, Technische Universität München, Germany; www.statistical-modelling.de/lst.html) (41). This toolbox was established on SPM12 based on the probabilistic modeling and the region growing algorithm. The algorithm first segmented the T1-weighted images into GM/ WM/ CSF. Lesion belief maps were created by combining these data with the intensities on FLAIR. By thresholding these maps with a pre-chosen initial threshold (κ), initial binary lesion maps were obtained, which were subsequently grown along the voxels that appeared hyperintense on the FLAIR image. We finally obtained the lesion probability maps and the total WMH volume of bilateral cerebral hemispheres (Figure 1).

Standardized WMH volume = actual WMH volume/ total intracranial volume (42). WMH volume (‰) was used to represent the standardized WMH volume in our study.

### Optical coherence tomography image acquisition and retinal thickness measure

**Figure 2:**
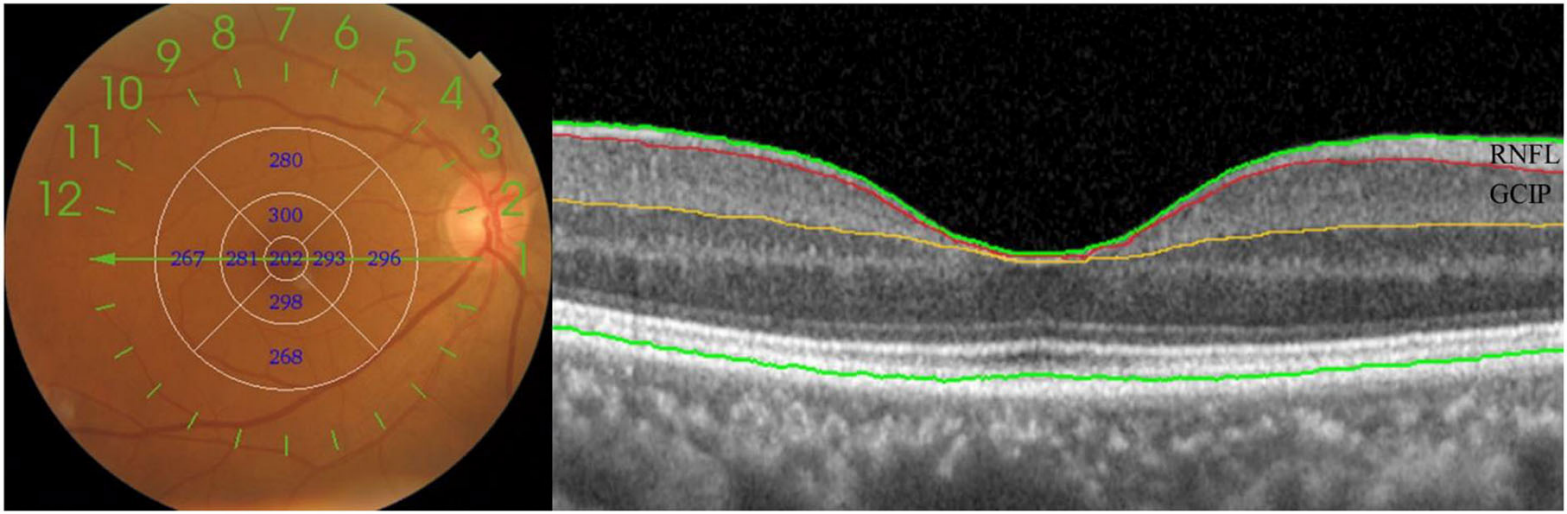
Optical coherence tomography of the retinal imaging. Nine subfields and retinal sublayers in macula. RNFL, retinal nerve fiber layer; GCIP, ganglion cell and inner plexiform layer.

Retinal images were taken for all subjects by the OCT imaging system. The Swept Source OCT (Topcon DRI OCT Triton, version 10.18, Topcon Corporation, Tokyo, Japan) with 3D scans (6.0 × 6.0 mm, 1,024 × 128) was used to obtain OCT scans centered at the fovea. The scans were obtained without pharmacological mydriasis under dark room conditions. All the OCT scans were reviewed by an experienced ophthalmologist to exclude poor-quality OCT scans. We excluded OCT scans of poor quality according to the OSCAR-IB criteria (43). The OCT scans were automatically segmented by the built-in software (Topcon Ophthalmic Data System IMAGEnet 6, version 1.2x, Topcon Corporation, Tokyo, Japan). According to the early treatment diabetic retinopathy study (ETDRS) grids, nine areas were divided by three concentric circles (diameter: 1 mm, 3 mm, and 6 mm) and lines in the “± 45 degree” direction. The thickness of the total retina, RNFL, and GCIP in inner/ outer superior, inner/ outer nasal, inner/ outer inferior, and inner/ outer temporal quadrants were recorded for each participant (Figure 2). Both eyes of each subject were scanned, and the average of both eyes was used in this study.

### Statistical analyses

**Figure 3:**
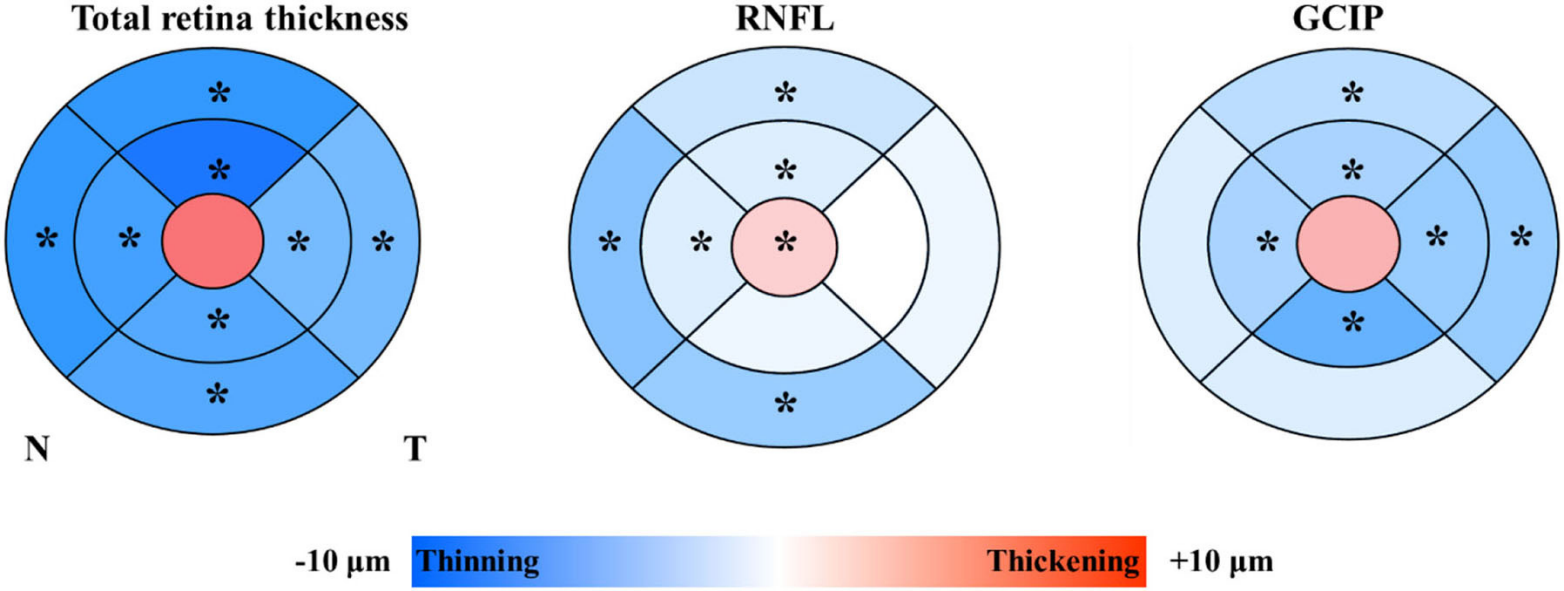
Colorimetric differences in different subfields between the lower white matter hyperintensity volume group and the higher white matter hyperintensity volume group. In red, thickening; in blue, thinning. RNFL, retinal nerve fiber layer; GCIP, ganglion cell and inner plexiform layer. *P < 0.05.

Continuous variables were presented as mean ± standard deviation or median (interquartile range) as appropriate. Categorical variables were presented as frequencies and percentages. The *T*-tests or Mann–Whitney *U*-tests were used for continuous variables, and χ^2^-tests were used for categorical variables. The thickness of the retina was analyzed by the *T*-tests. The pattern diagrams of ETDRS grids were drawn to display the changes in retina thickness more vividly (Figure 3). Thickening of the retina was shown in red and thinning was shown in blue, with the color strengthening as the change increased (15). The association between retinal thickness and WMH volume was investigated by binary logistic regression. A test for linear trend was conducted with the use of quartiles of the retinal thickness variables, with *P* < 0.1 in the binary logistic regression, as continuous variables by assigning the medians of the quartiles to the variables. The association between retinal thickness and WMH volume was further tested using the Pearson correlation analysis. SPSS version 26.0 (IBM Corporation, Armonk, New York, USA) was used for all analyses, with a *P-*value of < 0.05 as the significance level.

## Results

### Participant characteristics

We included 185 participants in this research. The participants were divided into two groups by the WMH volume (‰, standardized WMH volume) median. A total of 92 participants were grouped as the lower WMH volume group [WMH volume < 0.9496‰, mean age = 58.51 (9.18) years, male = 43.3%], and 93 participants were grouped as the higher WMH volume group [WMH volume ≥ 0.9496‰, mean age = 68.14 (10.05) years, male = 58.1%].

The characteristics of participants in the lower WMH volume group and the higher WMH volume group are summarized in Table 1. Individuals in the higher WMH volume group were significantly older, more likely to be male, and have a history of hypertension, CHD, and stroke than those in the lower WMH volume group. The higher WMH volume group presented higher SBP but lower LDL-C than the lower WMH volume group.

**Table 1:** Comparison of baseline characteristics between the lower white matter hyperintensity volume group and the higher white matter hyperintensity volume group.

	**Lower WMH volume group (*n* = 92)**	**Higher WMH volume group** **(*n* = 93)**	***P-*value**
Age, mean (SD), years	58.51 ± 9.18	68.14 ± 10.05	<0.001^*^
Gender (male), *n* (%)	40 (43.5)	54 (58.1)	0.047^*^
BMI, mean (SD), kg/m^2^	26.09 ± 3.7	25.52 ± 2.84	0.246
Hypertension, *n* (%)	40 (43.5)	67 (72)	<0.001^*^
Diabetes, *n* (%)	23 (25)	32 (34.4)	0.162
CHD, *n* (%)	9 (9.8)	22 (23.7)	0.012^*^
Stroke, *n* (%)	10 (10.9)	30 (32.3)	<0.001^*^
Current smoking, *n* (%)	17 (18.5)	19 (20.4)	0.737
Alcohol use, *n* (%)	10 (10.9)	15 (16.1)	0.295
SBP, median (IQR), mmHg	132.5 (122.25–143.75)	140 (127–156)	0.001^*^
DBP, median (IQR), mmHg	80 (75–86)	80 (73–88)	0.952
TC, mean (SD), mmol/L	4.7 ± 1.2	4.43 ± 1.26	0.139
TG, median (IQR), mmol/L	1.12 (0.89–1.61)	1.15 (0.85–1.57)	0.718
LDL-C, mean (SD), mmol/L	3.05 ± 0.84	2.77 ± 0.82	0.023^*^
HDL-C, median (IQR), mmol/L	1.11 (0.93–1.39)	1.17 (0.93–1.37)	0.663
FPG, median (IQR), mmol/L	5 (4.55–5.9)	5.28 (4.58–6.51)	0.425
UA, median (IQR), μmol/L	301.15 (234.55–353.18)	313 (262.35–364.8)	0.216
GM volume, mean (SD), ml	562.42 ± 59.36	551.38 ± 53.4	0.185
WM volume, median (IQR), ml	508 (468.25–547.75)	497 (463–528)	0.086

WMH, white matter hyperintensity; SD, standard deviation; BMI, body mass index; CHD, coronary heart disease; SBP, systolic blood pressure; IQR, interquartile range; DBP, diastolic blood pressure; TC, total cholesterol; TG, triglyceride; LDL-C, low density lipoprotein cholesterol; HDL-C, high density lipoprotein cholesterol; FPG, fasting plasma glucose; UA, uric acid; GM, gray matter; WM, white matter.

* Bold values indicate *P* < 0.05.

### Comparison of the retinal thickness in different subfields between the lower white matter hyperintensity volume group and the higher white matter hyperintensity volume group

Participants with higher WMH volume showed significantly thicker RNFL at the central fovea (*P* = 0.016), and thinner superior inner total retina (*P* < 0.001)/ RNFL (*P* = 0.034)/ GCIP (*P* = 0.014), nasal inner total retina (*P* = 0.006)/ RNFL (*P* = 0.039)/ GCIP (*P* = 0.027), inferior inner total retina (*P* = 0.007)/ GCIP (*P* = 0.018), temporal inner total retina (*P* = 0.017)/ GCIP (*P* = 0.033), superior outer total retina (*P* = 0.001)/ RNFL (*P* = 0.007) /GCIP (*P* = 0.006), nasal outer total retina (*P* < 0.001)/ RNFL (*P* < 0.001), inferior outer total retina (*P* = 0.003)/ RNFL (*P* = 0.001), and temporal outer total retina (*P* = 0.006)/ GCIP (*P* = 0.004) thickness, when compared to the participants with lower WMH volume (Table 2, Figure 3). No significant difference was seen in the thickness of total retina (*P* = 0.146)/ GCIP (*P* = 0.078) at the central fovea, inferior inner RNFL (*P* = 0.196), temporal inner RNFL (*P* = 0.972), nasal outer GCIP (*P* = 0.085), inferior outer GCIP (*P* = 0.105), and temporal outer RNFL (*P* = 0.188) when both groups were compared (Table 2, Figure 3).

**Table 2:** Comparison of the retinal thickness in different subfields between the lower white matter hyperintensity volume group and the higher white matter hyperintensity volume group.

	**Lower WMH volume group (*n* = 92)**	**Higher WMH volume group** **(*n* = 93)**	***P-*value**
**Macular central fovea**			
Retina, total, μm	235.42 ± 22.52	240.49 ± 24.63	0.146
RNFL, μm	8.17 ± 4.3	9.9 ± 5.38	0.016^*^
GCIP, μm	46.04 ± 10.77	48.99 ± 11.79	0.078
**Superior inner macula**			
Retina, total, μm	301.04 ± 15.55	291.26 ± 19.69	<0.001^*^
RNFL, μm	31.83 ± 7.38	29.6 ± 6.8	0.034^*^
GCIP, μm	84.7 ± 9.23	80.89 ± 11.52	0.014^*^
**Nasal inner macula**			
Retina, total, μm	301.12 ± 17.24	292.91 ± 22.27	0.006^*^
RNFL, μm	29.59 ± 8.28	27.23 ± 7.14	0.039^*^
GCIP, μm	83.76 ± 10.48	80.04 ± 12.19	0.027^*^
**Inferior inner macula**			
Retina, total, μm	294.44 ± 17.22	286.6 ± 21.87	0.007^*^
RNFL, μm	30.19 ± 9.53	28.47 ± 8.44	0.196
GCIP, μm	85.33 ± 26.88	77.91 ± 13.14	0.018^*^
**Temporal inner macula**			
Retina, total, μm	286.73 ± 16.23	279.89 ± 22.03	0.017^*^
RNFL, μm	25.3 ± 8.43	25.26 ± 9.73	0.972
GCIP, μm	78.23 ± 10.68	74.19 ± 14.59	0.033^*^
**Superior outer macula**			
Retina, total, μm	266.19 ± 16.17	257.48 ± 18.09	0.001^*^
RNFL, μm	44.18 ± 6.65	41.33 ± 7.51	0.007^*^
GCIP, μm	62.72 ± 6.89	59.36 ± 9.32	0.006^*^
**Nasal outer macula**			
Retina, total, μm	281.77 ± 15.76	272.99 ± 17.55	<0.001^*^
RNFL, μm	54.76 ± 10.5	49.3 ± 9.42	<0.001^*^
GCIP, μm	67.15 ± 8.16	65.09 ± 8.05	0.085
**Inferior outer macula**			
Retina, total, μm	257.66 ± 16.63	250.07 ± 17.34	0.003^*^
RNFL, μm	45.82 ± 7.46	41.77 ± 8.43	0.001^*^
GCIP, μm	61.29 ± 8.17	59.18 ± 9.4	0.105
**Temporal outer macula**			
Retina, total, μm	252.69 ± 16.83	245.73 ± 16.97	0.006^*^
RNFL, μm	28.22 ± 8.31	26.53 ± 9.02	0.188
GCIP, μm	66.15 ± 7.81	62.09 ± 10.82	0.004^*^

These values represent the mean with the SD. WMH, white matter hyperintensity; RNFL, retinal nerve fiber layer; GCIP, ganglion cell and inner plexiform layer.

* Bold values indicate *P* < 0.05.

### Association between the retinal thickness in different subfields and the risk of higher white matter hyperintensity volume

**Figure 4:**
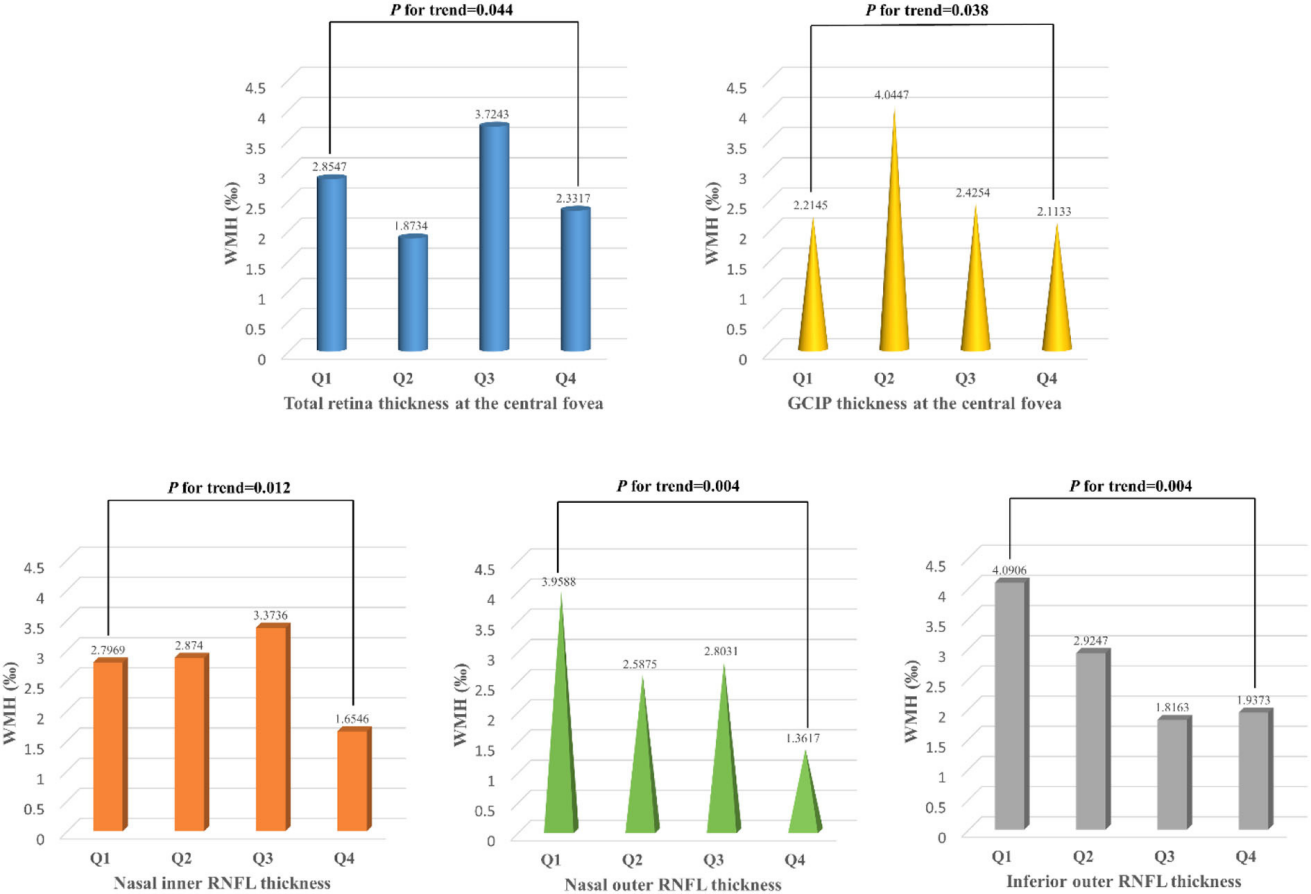
White matter hyperintensity volume of participants with different quartiles of the total retina and GCIP thickness at the central fovea, nasal inner RNFL, nasal outer RNFL, and inferior outer RNFL thickness. WMH, white matter hyperintensity; RNFL, retinal nerve fiber layer; GCIP, ganglion cell and inner plexiform layer. Total retinal thickness (*P* for trend = 0.044) and GCIP thickness (*P* for trend = 0.038) at the central fovea were positively associated with the risk of higher WMH volume, while nasal inner RNFL (*P* for trend = 0.012), nasal outer RNFL (*P* for trend = 0.004), and inferior outer RNFL (*P* for trend = 0.004) thickness were negatively associated with the risk of higher WMH volume.

**Figure 5:**
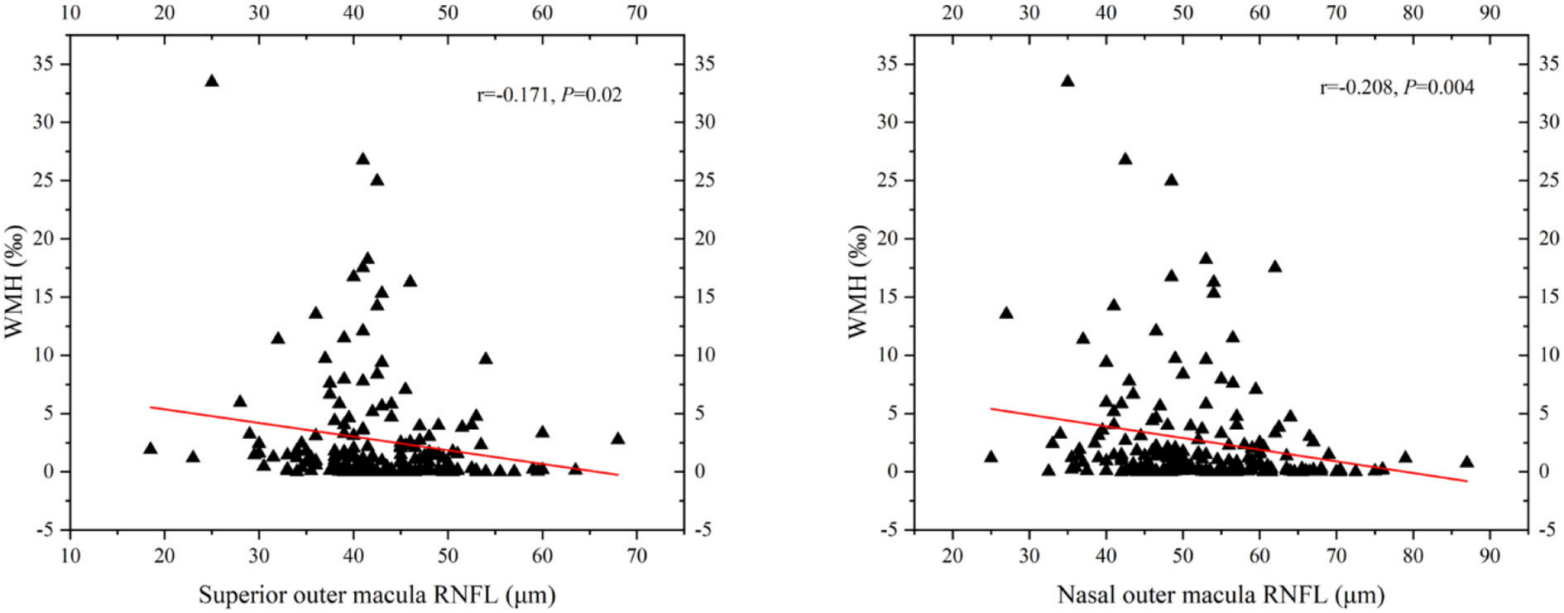
Scatterplot demonstrating the relationship between RNFL thickness and WMH volume (‰). WMH, white matter hyperintensity; RNFL, retinal nerve fiber layer. WMH volume had significant negative correlation with superior outer RNFL thickness (*r* = −0.171, *P* = 0.02) and nasal outer RNFL thickness (*r* = −0.208, *P* = 0.004).

In binary logistic regression, thicker total retina (OR: 1.018; 95% CI: 1.002 to 1.035; *P* = 0.026)/ GCIP (OR: 1.034; 95% CI: 1 to 1.068; *P* = 0.048) at the central fovea, and thinner nasal inner RNFL (OR: 0.952; 95% CI: 0.907 to 0.999; *P* = 0.048), nasal outer RNFL (OR: 0.937; 95% CI: 0.901 to 0.976; *P* = 0.002), inferior outer RNFL (OR: 0.933; 95% CI: 0.887 to 0.981; *P* = 0.006), and temporal outer GCIP (OR: 0.952; 95% CI: 0.908 to 0.997; *P* = 0.039) were associated with an increase in the risk of higher WMH volume, after adjusting for age, gender, hypertension, CHD, stroke, SBP, and LDL-C (Table 3). In the quartile-stratified analysis, we found that the risk of higher WMH volume showed a positive linear trend correlation with total retina and GCIP thickness at the central fovea, and a negative linear trend correlation with nasal inner RNFL, nasal outer RNFL, and inferior outer RNFL thickness (Table 4, Figure 4). The ORs of higher WMH volume of the highest quartile of retinal thickness compared with the lowest were 2.442 (95% CI: 0.848–7.034; *P* for trend = 0.044) for total retina thickness at the central fovea, 3.65 (95% CI: 1.263–10.549; *P* for trend = 0.038) for GCIP thickness at the central fovea, 0.26 (95% CI: 0.086–0.787; *P* for trend = 0.012) for nasal inner RNFL thickness, 0.181 (95% CI: 0.058–0.561; *P* for trend = 0.004) for nasal outer RNFL thickness, and 0.232 (95% CI: 0.081–0.667; *P* for trend = 0.004) for inferior outer RNFL thickness, after adjusting for age, gender, hypertension, CHD, stroke, SBP, and LDL-C. The correlation analysis results showed that WMH volume had a significantly negative correlation with superior outer RNFL thickness (*r* = −0.171, *P* = 0.02) and nasal outer RNFL thickness (*r* = −0.208, *P* = 0.004; Table 5, Figure 5).

**Table 3:** Logistic regression analysis of retinal thickness in different subfield-related factors for higher white matter hyperintensity volume.

	**OR (95% CI)**	***P-*value**
**Macular central fovea**		
Retina, total	1.018 (1.002–1.035)	0.026^*^
RNFL	1.074 (0.998–1.155)	0.056
GCIP	1.034 (1–1.068)	0.048^*^
**Superior inner macula**		
Retina, total	0.986 (0.966–1.007)	0.195
RNFL	0.969 (0.921–1.018)	0.208
GCIP	0.979 (0.944–1.015)	0.244
**Nasal inner macula**		
Retina, total	0.997 (0.973–1.011)	0.376
RNFL	0.952 (0.907–0.999)	0.048^*^
GCIP	0.99 (0.959–1.022)	0.546
**Inferior inner macula**		
Retina, total	0.993 (0.974–1.012)	0.438
RNFL	0.975 (0.938–1.013)	0.194
GCIP	0.978 (0.948–1.01)	0.178
**Temporal inner macula**		
Retina, total	0.988 (0.97–1.007)	0.214
RNFL	0.999 (0.961–1.037)	0.939
GCIP	0.976 (0.946–1.007)	0.124
**Superior outer macula**		
Retina, total	0.987 (0.965–1.009)	0.239
RNFL	0.955 (0.907–1.006)	0.08
GCIP	0.96 (0.915–1.008)	0.102
**Nasal outer macula**		
Retina, total	0.995 (0.972–1.018)	0.665
RNFL	0.937 (0.901–0.976)	0.002^*^
GCIP	1.023 (0.978–1.071)	0.32
**Inferior outer macula**		
Retina, total	1.001 (0.978–1.024)	0.965
RNFL	0.933 (0.887–0.981)	0.006^*^
GCIP	1.007 (0.964–1.053)	0.784
**Temporal outer macula**		
Retina, total	0.993 (0.97–1.017)	0.546
RNFL	0.986 (0.946–1.027)	0.49
GCIP	0.952 (0.908–0.997)	0.039^*^

Adjusted for age, gender, hypertension, coronary heart disease, stroke, systolic blood pressure, and low-density lipoprotein cholesterol. RNFL, retinal nerve fiber layer; GCIP, ganglion cell and inner plexiform layer.

* Bold values indicate *P* < 0.05.

**Table 4:** ORs (and 95% CIs) of higher white matter hyperintensity volume by quartiles of retinal thickness in different subfields.

	**Quartiles of retinal thickness**				
	**1**	**2**	**3**	**4**	***P*-trend**
**Macular central fovea**					
**Retina, total**					
Median, μm	211.5	229	243	268	
OR (95% CI)	1 (Ref)	0.962 (0.343–2.693)	2.779 (0.949–8.137)	2.442 (0.848–7.034)	0.044^*^
**RNFL**					
Median, μm	4.5	6.5	10	15	
OR (95% CI)	1 (Ref)	1.011 (0.386–2.649)	0.756 (0.272–2.098)	2.412 (0.882–6.593)	0.083
**GCIP**					
Median, μm	36.25	43.25	49.5	61	
OR (95% CI)	1 (Ref)	2.301 (0.818–6.474)	1.421 (0.525–3.848)	3.65 (1.263–10.549)	0.038^*^
**Nasal inner macula**					
**RNFL**					
Median, μm	22	25	29.5	36.5	
OR (95% CI)	1 (Ref)	1.009 (0.374–2.725)	0.892 (0.324–2.452)	0.26 (0.086–0.787)	0.012^*^
**Superior outer macula**					
**RNFL**					
Median, μm	36	41	45	50.75	
OR (95% CI)	1 (Ref)	0.815 (0.295–2.25)	1.147 (0.424–3.099)	0.445 (0.169–1.176)	0.145
**Nasal outer macula**					
**RNFL**					
Median, μm	41	48	55	64.25	
OR (95% CI)	1 (Ref)	0.83 (0.303–2.276)	0.833 (0.299–2.321)	0.181 (0.058–0.561)	0.004^*^
**Inferior outer macula**					
**RNFL**					
Median, μm	36	41	46	52.5	
OR (95% CI)	1 (Ref)	0.754 (0.272–2.089)	0.447 (0.165–1.214)	0.232 (0.081–0.667)	0.004^*^
**Temporal outer macula**					
**GCIP**					
Median, μm	54.75	63	67.5	74	
OR (95% CI)	1 (Ref)	0.705 (0.263–1.892)	0.501 (0.18–1.392)	0.418 (0.144–1.214)	0.09

Adjusted for gender, age, hypertension, coronary heart disease, stroke, systolic blood pressure, and low-density lipoprotein cholesterol. RNFL, retinal nerve fiber layer; GCIP, ganglion cell and inner plexiform layer.

* Bold values indicate *P* < 0.05.

**Table 5:** Correlation analysis between white matter hyperintensity volume and retinal thickness.

	***r***	***P-*value**
**Macular central fovea**		
Retina, total	0.005	0.941
RNFL	0.066	0.372
GCIP	−0.02	0.79
**Nasal inner macula**		
RNFL	−0.103	0.163
**Superior outer macula**		
RNFL	−0.171	0.02^*^
**Nasal outer macula**		
RNFL	−0.208	0.004^*^
**Inferior outer macula**		
RNFL	−0.144	0.051
**Temporal outer macula**		
GCIP	−0.144	0.051

RNFL, retinal nerve fiber layer; GCIP, ganglion cell and inner plexiform layer.

* Bold values indicate *P* < 0.05.

### Association between the retinal thickness in different subfields and the risk of moderate-severe periventricular and deep white matter hyperintensity

We divided the participants into the mild group (Fazekas 0–1) and the moderate-severe group (Fazekas 2–3) according to the PWMH score and DWMH score, respectively. In binary logistic regression, thicker total retina (OR: 1.017; 95% CI: 1.001–1.033; *P* = 0.039)/ RNFL (OR: 1.089; 95% CI: 1.009–1.175; *P* = 0.028) at the central fovea and thinner nasal outer RNFL (OR: 0.958; 95% CI: 0.924–0.994; *P* = 0.024) were associated with an increase in the risk of moderate-severe PWMH, while thinner superior outer RNFL (OR: 0.94; 95% CI: 0.891–0.991; *P* = 0.023), nasal outer RNFL (OR: 0.935; 95% CI: 0.899–0.973; *P* = 0.001), inferior outer RNFL (OR: 0.926; 95% CI: 0.881–0.974; *P* = 0.003), and temporal outer GCIP (OR: 0.921; 95% CI: 0.879–0.966; *P* = 0.001) were associated with an increase in the risk of moderate-severe DWMH, after adjusting for age, gender, hypertension, CHD, stroke, SBP, and LDL-C (Table 6).

**Table 6:** Logistic regression analysis of retinal thickness in different subfield-related factors for moderate-severe periventricular and deep white matter hyperintensity.

	**PWMH**		**DWMH**	
	**OR (95% CI)**	***P-*value**	**OR (95% CI)**	***P-*value**
**Macular central fovea**				
Retina, total	1.017 (1.001–1.033)	0.039^*^	0.999 (0.985–1.014)	0.902
RNFL	1.089 (1.009–1.175)	0.028^*^	1.019 (0.953–1.089)	0.58
GCIP	1.033 (0.999–1.067)	0.056	0.974 (0.944–1.006)	0.107
**Nasal inner macula**				
RNFL	0.974 (0.932–1.017)	0.236	0.961 (0.916–1.008)	0.103
**Superior outer macula**				
RNFL	0.951 (0.904–1.001)	0.053	0.94 (0.891–0.991)	0.023^*^
**Nasal outer macula**				
RNFL	0.958 (0.924–0.994)	0.024^*^	0.935 (0.899–0.973)	0.001^*^
**Inferior outer macula**				
RNFL	0.959 (0.918–1.002)	0.06	0.926 (0.881–0.974)	0.003^*^
**Temporal outer macula**				
GCIP	0.987 (0.946–1.03)	0.559	0.921 (0.879–0.966)	0.001^*^

Adjusted for gender, age, hypertension, coronary heart disease, stroke, systolic blood pressure, and low-density lipoprotein cholesterol. PWMH, periventricular white matter hyperintensity; DWMH, deep white matter hyperintensity; RNFL, retinal nerve fiber layer; GCIP, ganglion cell and inner plexiform layer.

* Bold values indicate *P* < 0.05.

## Discussion

We investigated the relationship between the retinal thickness in different subfields and the WMH volume in this study. We found that the risk of higher WMH volume showed a positive linear trend correlation with total retina and GCIP thickness at the central fovea, and a negative linear trend correlation with nasal inner, nasal outer, and inferior outer RNFL thickness. WMH volume had a significantly negative correlation with superior outer and nasal outer RNFL thickness. In addition, DWMH was more correlated with the thinning of outer RNFL, while PWMH was more correlated with the thickening of the retina at the central fovea. Our results further verified the potential association between the retina and the brain. Retinal thickness measured by OCT may become a non-invasive, affordable, and convenient indicator for WMH.

The association between thinner RNFL and higher WMH volume may be due to the mechanism of chronic ischemia. Researchers showed that vascular risk factors (e.g., diabetes, hypertension, anemia, carotid plaque, dyslipidemia, and inflammatory cytokine level) leading to reduced blood flow, tissue ischemia, and hypoxia could also cause retinal changes and ultimately affect vision, which is similar to the pathological mechanism of WMH (12, 44). OCTA is a novel technique used to image retinal microvascular impairment rapidly and non-invasively. Increasing evidence shows that microvascular density evaluated by OCTA is associated with the degree of WMH, notably the DWMH, which is related to the thinning of RNFL (29, 34). It suggests that microvascular damage occurs in both the retina and brain, results in impaired neurovascular coupling, and eventually leads to nerve fiber lesions (30, 31, 45). Furthermore, retrograde neuroaxonal degeneration is considered to be another possible explanation for the thinning of RNFL (46). However, more and more reports have indicated that retinal neurodegeneration may precede microvascular damage caused by brain-related diseases, and could lead to a secondary decrease in blood flow density (30, 47). Its mechanism is still unclear, and further studies are needed. In summary, RNFL thickness changes are expected to be one of the biological markers to reflect the axonal damage and DWMH caused by vascular factors in the super-early stage.

In this study, WMH volume had a significant negative correlation with superior outer and nasal outer RNFL thickness, rather than in the other quadrants. The precise cause of the area difference in RNFL thickness reduction is unknown at this time. It might be due to the special direction of the nerve fibers in the macula. Nerve fibers in the nasal quadrant arrive at the optic disk directly. Nerve fibers in the temporal quadrant are bounded by horizontal meridians, which, respectively, curve around the macular fovea and reach the optic disk (10). Among healthy individuals, the retinal thickness in the nasal quadrant is the thickest and the temporal quadrant is the thinnest (48). There are more nerve fibers in the nasal quadrant, whose demand for oxygen and nutrients is larger. As a result, nerve fibers in the nasal quadrant are more prone to ischemia and hypoxia than in the temporal quadrant. Similarly, previous studies showed that DWMH was more related to ischemic factors (6). On the contrary, in the temporal quadrant, axons are not arranged with such a high density. They usually run among ganglion cells, not constituting an independent layer. The thin hyperreflective band observed in the temporal quadrant may not truly reflect the thickness of RNFL (9). This may explain the significant reduction of GCIP, rather than RNFL, found in the temporal outer macula in the higher WMH group.

Our research presented that the thickening of the total retina and GCIP at the central fovea significantly increased the risk of higher WMH volume. We suspect that this is associated with neural inflammation. Multiple studies have shown that the levels of inflammatory factors were positively correlated with retina thickness at the central fovea (49–51). Cytokines could damage the connexins between endothelial cells, increase the permeability of vessels, and induce leukocyte aggregation. It would break the blood–retinal barrier (52, 53). Proteins, liquids, and other substances accumulate in the retina, resulting in its thickening. Cytokines activate microglia and astrocytes, further promoting inflammatory response and leading to neuronal dysfunction and apoptosis (54, 55). This process is similar to the mechanism of PWMH. In addition, several studies showed that inflammatory response mainly affected the thickness of macular fovea, which might be due to Müller cells. Müller cells are responsible for the functional and metabolic support of their associated neurons and provide neurons with trophic substances and metabolic waste removal (55). In the macular fovea area, Müller cell density is five times higher when compared to the periphery. Müller cells are highly elongated in this area and follow a “*Z*-shape” trajectory to extend around (53). Their prolongations are closely bound to neuronal axons by junction proteins, as a “molecular filter” to prevent macromolecules from aggregating in the retina (56). When junction proteins are broken, proteins and other substances would leak into the retina (55). Furthermore, abnormal aquaporin (AQP) in Müller cells also affects local retinal thickness. AQP-4 is highly expressed in the glial cells of the brain. It constitutes a “glymphatic pathway” for the waste clearance of the brain (45). Similarly, AQP-4 expressed along macular Müller cells also constitutes a “glymphatic pathway” to transport proteins to the retina (57). The disorder of AQP-4 in Müller cells contributes to the drainage dysfunction in the macular area and the swelling of cells. Differently, studies have found that there was a defect in the blood–retinal barrier at the optic disk allowing the unobstructed drainage of proteins to prevent inflammatory edema (58). Interestingly, PWMH is also closely related to inflammatory factors and is more edematous compared to DWMH (7). In conclusion, changes in macular fovea thickness may offer a new view in understanding the pathology of WMH caused by inflammatory factors.

Another possible explanation for the relationship between the thickening of the central fovea and higher WMH volume might be the deposition of amyloid-β (Aβ). Studies found that Aβ deposits may occur in the retina, even before the deposits in the brain (59). Bernardes et al. reported that the mice harboring three human mutant genes of AD showed a thicker RNFL + GCL until 4 months old (48). Aβ deposits may contribute to the inflammatory processes, neuronal degeneration, and aging in the retina and finally change retinal thickness (60, 61).

Similar observations have documented that the thinning of RNFL was related to the degree of WMH (30, 33, 34, 62). However, a prospective study unexpectedly showed that WMH aggravation was not related to the decrease in RNFL thickness (63). As for GCIP, the study of Qu et al. presented reduced GCIP in the hypertensive WMH group, which was inconsistent with our results (33). We speculate that this difference may be due to different retinal zoning methods, short follow-up, and limited sample capacity. Unlike these studies, we skillfully divided the macular retina into nine subfields, considering the different cell distributions in different retinal regions. Moreover, we measured the volume of WMH for a more scientific and rigorous reflection of the WMH state.

Retinal thickness changes are in many cases compensatory among subfields and sublayers. It is closely related to the measuring method. A previous study found that geometrically predetermined regions (e.g., concentric rings or ETDRS grids) might cause thickening and thinning within the same region to cancel each other out and lead to the clinically relevant differences being masked. In contrast, the color-coded quantitative maps could allow point-to-point comparisons between groups and provide more precise regions of change (59). Moreover, at the same point, the thickness of a certain layer would also affect the thickness of adjacent layers (59). The thickening of GCIP in this study cannot be excluded as the result of thinning of other contiguous layers. Therefore, quantitative maps of the whole retinal layers should be used to reflect the retinal changes in WMH individuals with greater accuracy.

There are several limitations to our study. First, retrospective studies can just investigate the associations. Prospective studies are necessary to demonstrate our results. Second, the sample capacity of this study is restricted. The hypothesis must be validated in a large study. Third, we did not measure the thickness of other retinal sublayers separately and RNFL in the peripapillary area, so we cannot explore their relationship with WMH and the compensatory among sublayers. Complementary studies where each retinal layer is studied separately, as well as the RNFL in the peripapillary area, are needed. Furthermore, PWMH and DWMH have different mechanisms, but the LST toolbox cannot distinguish between them. More sophisticated software is needed to measure WMH volume in different regions. Furthermore, we did not measure the thickness of the outer retinal sublayers separately, so we cannot explore the relationship between outer retinal sublayers and WMH. Further large prospective studies with more exact instruments to prove the longitudinal association between all retinal sublayers thickness and WMH are required to solve these problems.

## Conclusion

In conclusion, we found that the increase in the risk of higher WMH volume was associated with the thickening of total retina and GCIP at the central fovea, and the thinning of nasal inner, nasal outer, and inferior outer RNFL. WMH volume had a significant negative correlation with superior outer and nasal outer RNFL thickness. DWMH was more correlated with the thinning of outer RNFL, while PWMH was more correlated with the thickening of the retina at the central fovea. This study provides new evidence for the potential retina–brain association. OCT, as a non-invasive, reproducible, affordable, and convenient technique, may prove to be a biological marker for the early detection and longitudinal monitoring of WMH. Additionally, dynamic observation of retinal thickness changes could also be used to studies on the mechanism of some brain-related diseases. To fully elucidate the relationship between retinal thickness and WMH, further longitudinal and randomized studies with larger sample capacity are needed.

## Data availability statement

The raw data supporting the conclusions of this article will be made available by the authors, without undue reservation.

## Ethics statement

The studies involving human participants were reviewed and approved by Ethical Committee of Hebei General Hospital (approval number 2022105). Written informed consent for participation was not required for this study in accordance with the national legislation and the institutional requirements. Written informed consent was not obtained from the individual(s) for the publication of any potentially identifiable images or data included in this article.

## Author contributions

PL proposed the conception of the study and revised the manuscript. XL performed the data acquisition and wrote the manuscript. ZT contributed to the analysis and interpretation of data. ZJ, YD, and JX offered clinical suggestions and performed the data validation. All authors contributed to the article and approved the submitted version.

## Conflict of interest

The authors declare that the research was conducted in the absence of any commercial or financial relationships that could be construed as a potential conflict of interest.

## Publisher's note

All claims expressed in this article are solely those of the authors and do not necessarily represent those of their affiliated organizations, or those of the publisher, the editors and the reviewers. Any product that may be evaluated in this article, or claim that may be made by its manufacturer, is not guaranteed or endorsed by the publisher.
